# Breast Tissue Metabolism by Magnetic Resonance Spectroscopy

**DOI:** 10.3390/metabo7020025

**Published:** 2017-06-07

**Authors:** Naranamangalam R. Jagannathan, Uma Sharma

**Affiliations:** Department of NMR & MRI Facility, All India Institute of Medical Sciences, New Delhi 110029, India; umasharma69@aiims.edu

**Keywords:** magnetic resonance spectroscopy (MRS), breast cancer, tissue metabolism, in vivo, in vitro, High-resolution magic angle spinning (HRMAS)

## Abstract

Metabolic alterations are known to occur with oncogenesis and tumor progression. During malignant transformation, the metabolism of cells and tissues is altered. Cancer metabolism can be studied using advanced technologies that detect both metabolites and metabolic activities. Identification, characterization, and quantification of metabolites (metabolomics) are important for metabolic analysis and are usually done by nuclear magnetic resonance (NMR) or by mass spectrometry. In contrast to the magnetic resonance imaging that is used to monitor the tumor morphology during progression of the disease and during therapy, in vivo NMR spectroscopy is used to study and monitor tumor metabolism of cells/tissues by detection of various biochemicals or metabolites involved in various metabolic pathways. Several in vivo, in vitro and ex vivo NMR studies using ^1^H and ^31^P magnetic resonance spectroscopy (MRS) nuclei have documented increased levels of total choline containing compounds, phosphomonoesters and phosphodiesters in human breast cancer tissues, which is indicative of altered choline and phospholipid metabolism. These levels get reversed with successful treatment. Another method that increases the sensitivity of substrate detection by using nuclear spin hyperpolarization of ^13^C-lableled substrates by dynamic nuclear polarization has revived a great interest in the study of cancer metabolism. This review discusses breast tissue metabolism studied by various NMR/MRS methods.

## 1. Introduction

The second leading cause of death in women worldwide is breast cancer, due to its high incidences of mortality and morbidity. It is known that during cancer progression, the metabolism of cells or tissues is altered and some of these metabolic alterations are linked to drug resistance. In fact, a complex network of interacting genes, proteins and metabolite reactions in various metabolic pathways take place in an organized and regulated manner in normal cells [1]. However, during malignancy, many of these regulatory pathways are dysregulated, leading to uncontrolled growth and proliferation [1,2] and the cells adopt alternative metabolic pathways [3]. These processes are not caused by any one single event, but by multiple events. Furthermore, the microenvironment and physiological environments (such as hypoxia and acidic extracellular pH) also influence tumor metabolism.

Breast cancer is a clinically heterogeneous disease and factors including hormone receptor status, lymph node involvement, and the grade of the tumor can influence the patient’s outcome. Classification is based on the analysis of tumor morphology and histopathological detection of estrogen receptor (ER), progesterone receptor (PR) and human epidermal growth factor receptor 2 (HER2). These molecular features play an important role in making the therapeutic decision. The treatment regime of a particular patient is based on the molecular profile, characteristics of the cancer and how the drugs can target metabolic pathways. In this direction, non-invasive magnetic resonance (MR) based techniques play an important role in the study of tumor tissue metabolism. In vivo ^1^H and ^31^P MRS studies on breast cancers have documented increased levels of total choline (tCho), phosphomonoesters (PME), and phosphodiesters (PDE) as metabolic features of cancer [4,5]. In fact, studies have indicated that the metabolic hallmark of cancer is the abnormal choline and phospholipid metabolism that is associated with oncogenesis and tumor progression [6,7,8,9,10]. Upon successful therapy, the levels of these metabolites (tCho, PME and PDE) may be lowered and hence are being considered as biomarkers for monitoring the tumor response [11,12]. The literature on breast cancer metabolism using MR is vast, with studies (in vivo, in vitro and ex vivo) on cells, extracts, xenografts, and tissues. Thus, it is not our intent to review all these studies, but to focus on the metabolism studied using NMR/MRS in breast tissues. However, the results from the cell-line studies on breast cancer are important to explain and understand the many metabolic features that are observed in ex vivo and in vivo tissues.

## 2. MR Methodologies to Study Breast Tissue Metabolism

There are various MR methodologies used to study tumor tissue metabolism. The first is magnetic resonance imaging (MRI), the most sensitive imaging modality that presents excellent images of soft tissue. The introduction of MRI in clinical medicine has revolutionized patient management by aiding the diagnosis, pre-operative cancer staging, definition of the extent of the disease, and therapeutic monitoring. A clinical radiologist, besides using the tumor size, also reports qualitative and semi-quantitative characteristics based on a Breast Imaging Reporting and Data System (BI-RADS) score [13].

Secondly, dynamic contrast-enhanced MRI provides both morphological and functional information with good sensitivity but variable specificity. Studies have also shown that malignant breast tissue enhances more rapidly compared to normal breast tissue following the administration of a contrast agent such as gadolinium chelate. Also, methods that use the differences in various biophysical, biochemical, and physiological characteristics of malignant, benign, and normal breast tissues have been explored. These include the diffusion of water molecules (diffusion-weighted imaging), vascularity (perfusion weighted imaging), and biochemical/metabolite markers (MR spectroscopy). Diffusion MRI provides information on the extracellular and intracellular tissue compartments as well as information on the altered metabolism during malignant transformation. During cancer growth, the presence of cell membranes, macromolecules, and organelles restrict the motion of water molecules and therefore decreases the diffusion constant of water compared to the mobility of the water molecules in normal tissues. This is represented as the apparent diffusion coefficient (ADC). ADC has been used in differentiating various tissue pathologies and diffusion MRI has, in fact, been established as an important adjunct technique with many clinical applications. The utility of diffusion MRI in the differentiation of malignant and benign breast tissues as well as in the monitoring of the treatment response has been well documented [14]. Studies have also documented a relationship between the cell density and ADC.

In vivo magnetic resonance spectroscopy (MRS), a non-invasive modality, provides an insight into the underlying biochemical processes associated with the malignant transformation of normal cells or tissue. MR imaging presents tumor morphology, while MR spectroscopy provides the biochemical information for understanding the disease’s physiology and metabolism [15]. The chemical composition from a well-defined region of interest (ROI) or volume of interest in any organ of the human body can be obtained using in vivo MRS. The breast tissue biochemistry, using proton (^1^H) and phosphorous (^31^P) nuclei due to their high natural abundance and sensitivity, has been the focus in the majority of in vivo MRS studies. The compounds determined by MRS also serve as biomarkers and are essential for the early diagnosis and for treatment monitoring. Further, by using high-resolution NMR it is possible to study intact tissues (ex vivo NMR or high-resolution magic angle spinning MRS, HRMAS), tissues taken out after surgery, fine needle aspiration biopsy or true cut biopsy samples (in vitro NMR), as well as bio-fluids. These investigations help us to study the molecular pathways and interactions through the detection and monitoring of small molecular compounds (metabolites). This area of metabolomics using NMR has the potential to give information of the compounds that are intermediate or end products in various ongoing biochemical processes, and which are important in the study of tissue metabolism. The following sections present a few examples of studies that were carried out using the above-mentioned MR methodologies to investigate breast tissue metabolism.

### 2.1. In vitro High-Resolution NMR Spectroscopy

In vitro high-resolution NMR spectroscopy is a powerful tool in evaluating tumor metabolism by the analysis of body fluids like urine, blood, and intact and extracted tissue specimens. Unlike in vivo MRS examination (vide infra), the in vitro NMR analysis of tissue and fluid samples is not limited by low spectral resolution and low sensitivity. In fact, the advantages of in vitro ^1^H MRS over in vivo MRS investigations are that the spectra can be acquired at very high magnetic fields with increased sensitivity and spectral resolution. This helps with the identification of a large number of metabolites and the absolute quantification of the metabolites, which facilitates the metabolomic analysis. A great deal of information can be obtained from the presence of various metabolites and other small organic molecules that can be correlated to various stages of cancer. Additionally, 2D NMR experiments are necessary for accurate assignment of metabolites and can be easily carried out using in vitro NMR of tissues, which may run for hours. Further, the motion artefacts of subjects normally seen with in vivo MRS are not present while examining fluid or tissue samples.

In most cases of in vitro NMR spectral analysis of tissues, extraction methods such as: (i) perchloric acid tissue extraction for water-soluble metabolites; (ii) chloroform and methanol extraction for lipid-soluble metabolites, are used. For the simultaneous extraction of both the aqueous and the lipid soluble metabolites, the methanol-chloroform-water extraction is used. In the extraction procedure, the macromolecules are removed to improve the spectral resolution of signals from the low molecular weight metabolites. However, it is possible that the extraction procedures used can cause decomposition of the tissue which may results in the loss of metabolites. Beckonert et al. have reviewed the NMR spectroscopic analysis of urine, plasma, serum, and tissue extracts [16].

**Figure 1:**
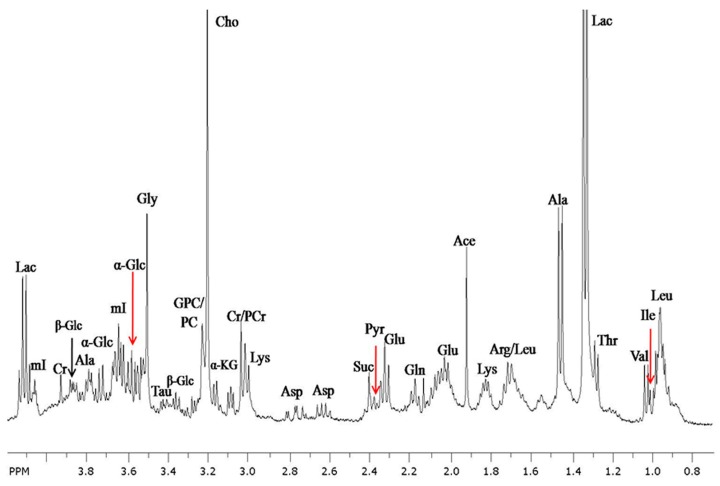
In vitro ^1^H magnetic resonance (MR) spectrum from the aliphatic region of the perchloric acid extracted from involved breast cancer tissue recorded at 400 MHz nuclear magnetic resonance (NMR). Abbreviations used: Ala, alanine; Ace, acetate; Arg, arginine; Asp, aspartate; Cho, choline; Cr, creatine; Glc, glucose; Glu, glutamate; Gln, glutamine; GPC, glycerophosphocholine; Gly, glycine; Iso, isoleucine; KG, ketogultarate Lac, lactate; Leu, leucine; Lys, lysine; mI, myo-inositol; PCr, phosphocreatine; PCho, phosphocholine; Pyr, pyruvate; Suc, succinate; Tau, taurine; Val, valine.

The first comprehensive in vitro NMR work was carried out by Gribbestad et al. [17] on normal and malignant breast tissues. A total of 30 metabolites were identified with significant differences in the level of several metabolites between involved and non-involved breast tissues. A study from our laboratory showed that tumor breast tissues have a significantly higher concentration of lactate, alanine, lysine, acetate, glutamic acid, glutamine, phosphocreatine+creatine, Cho, glycerophosphocholine (GPC)+ phosphocholine (PCho), and myo-inositol in comparison to non-involved tissues [18] (see Figure 1). No significant difference was seen in the concentration of tyrosine, phenylalanine, glucose, or formate between the invasive tumor and non-involved tissues.

Combined with the pattern-recognition and visualization methods, Beckonert et al. [19] reported an in vitro ^1^H MRS analysis of tissue extracts to monitor the metabolic differences between various tissue types. Increased uridine di-phosphate-hexose, phosphocholine, and phosphoethanolamine were seen according to the tumor grade. Taurine and lipid metabolites were higher in malignant samples. In control samples, myo-inositol and glucose were elevated in comparison to the malignant tissues. Both compounds also characterized different subgroups in the pool of unaffected breast tissue samples depending upon fat content or fibrosis.

Thus, the analysis of an in vitro NMR spectral profile of tissues can provide tumor biomarkers which can then be used for early diagnosis and clinical management. Additionally, the metabolites derived from in vitro NMR provide insights into the biochemical changes associated with the cancer and can give a better understanding of the tumor biology. In most cases, it is possible to compare the results of in vitro NMR studies of tissues with the in vivo MRS data to validate clinical MRS observations and to illustrate that these techniques are complimentary to each other.

### 2.2. High-Resolution Magic Angle Spinning (HRMAS) ^1^H NMR Spectroscopy

^1^H HRMAS NMR spectroscopy is the method of choice to study cells and intact tissue biopsy/surgery samples. This method also provides information on tissue metabolomics to help understand the metabolism of the breast tissue. The sample preparation requirements are minimal and the metabolic information obtained using HRMAS of intact tissues is closer to in vivo conditions since the native morphological structures of the tissues are preserved. Another advantage of HRMAS is that it allows the analyzed tissue to be further studied by histopathology and molecular biology analyses like immunohistochemistry, polymerase chain reaction, liquid chromatography–mass spectrometry etc. This means that both the histological and metabolic information are collected from the same tissue samples. However, the HRMAS method requires a specialized RF probe that is required to spin the sample (tissues) at a 54.7° angle to reduce the line-broadening effect that is normally observed in non-liquid samples, like tissues. This is due to the heterogeneity of the sample and the residual anisotropy that are generally averaged out in the solution. However, using the standard probes designed for solution-state NMR of samples, in vitro NMR can be performed. Unlike in vitro NMR which requires the extraction of metabolites (using perchloric acid, methanol, etc.), which can alter the nature of tissues; HRMAS NMR can be performed with intact tissues. However, the degradation of metabolites in the intact tissue during HRMAS experiments may be of concern. Recently, Fuss and Cheng have reviewed the role of cancer metabolism using ex vivo HRMAS [20].

HRMAS ^1^H MRS of intact breast cancer tissue specimens showed a highly resolved spectral profile with better resolution and immense diagnostic potential [21,22,23]. In these specimens, a number of resonances from amino acids (including alanine, asparagine, aspartate, histidine, leucine, isoleucine, glutamate, glutamine, glycine (Gly), lysine, tyrosine, and valine), energy metabolites (glucose and lactate), choline containing compounds (GPC, PCho and Cho), and lactate were observed. Metabolic markers like PCho, lactate, and lipids were quantified and correlated with the histopathological grade for improved accuracy of the diagnosis. Because breast cancer is highly heterogeneous, the metabolic profiles obtained by HRMAS are seen to be different in spectra from patients with similar diagnoses, especially in the region of lipid signals [22].

Further, the HRMAS ^1^H MRS of breast cancer tissues was also compared with the conventional high-resolution spectra of perchloric acid extracts of the same tissue type [23]. The spectral resolution obtained using HRMAS was found to be grossly comparable to the spectra of the extracts, and nearly 30 different metabolites could be assigned. The difference seen in the MR spectra of intact breast cancer tissue using HRMAS and the perchloric acid extracts is largely in the region of lipid resonances (due to adipose tissue present in the breast tissue) and in the low field region. The study of the HRMAS of breast cancer tissues with the non-involved adjacent tissue from the same patients has also been reported [24]. It is shown that tissues with a high fraction of tumor cells have a different metabolic profile than tissues with a low fraction of tumor cells. Also, high tumor fraction tissues show elevated lactate, creatine, Gly, and PC. Bathen et al. [25] reported multivariate models relating spectral data to the histological grade, lymphatic spread, and hormone status. They reported that patients with local spread to the axillary lymph nodes showed enhanced Gly and PCho and reduced betaine and taurine, compared to those without axillary lymph node spread. These results showed that a MR-determined metabolic phenotype may have a role in decision making for adjuvant chemotherapy [25].

Li et al. [26] showed a good discrimination between 13 cancer and 18 non-cancer samples (obtained by percutaneous core needle biopsy) using a multivariate model and HRMAS ^1^H MR data. A sensitivity of 69% with 94% specificity was achieved in predicting the cancer status. Metabolic profiling of core needle biopsy samples using HRMAS NMR to predict pathologic response to neoadjuvant chemotherapy in locally advanced breast cancer patients has also been reported [27]. The multivariate analysis of pretreatment core biopsy samples using choline containing metabolites showed that it is possible to predict the pathological response before the initiation of treatment. The prediction of prognostic factors, such as axillary lymph node and ER and PR receptor status, was studied in 160 surgically excised breast tissues using HRMAS [28]. ER and PR status were best predicted by Partial least squares Discriminant Analysis (PLS-DA) with a correct classification of 44 out of 50 and 39 out of 50 samples, respectively. Bayesian belief networks best predicted the lymph node status correctly in 34 out of 50 samples.

Guiskaodegard et al. [29] examined the relation between metabolite profiles obtained from surgical tumor biopsies and long-term (5-year) breast cancer survival. Breast cancer tissues excised during surgery from 98 patients were subjected HRMAS NMR and the data were analyzed by multivariate principal component analysis and PLS-DA. The predictions of 5-year survival were carried out using MRS data and compared with the predictions using clinical data. The results showed that 71 ER+ patients could be separated into two groups with significantly different survival rates. A lower survival rate was seen to be associated with elevated Gly and lactate, indicating that these metabolites may be useful as biomarkers for breast cancer prognosis. No such metabolic differences were observed for ER− patients with the survival rate.

It is known that lactate is increased under both aerobic and anaerobic conditions in many cancers relative to normal tissue. During hypoxia, because of decreased oxygen, adenosine triphosphate is generated by conversion of glucose to lactate via glycolysis [30]. In solid tumors hypoxia is common and has been correlated with aggressive and metastatic tumors [31]. Further, with sufficient oxygen levels, cancer cells may convert glucose to lactate (the Warburg effect) [32], but it is still not clear as to why this is so. It may be because proliferating cells have higher metabolic requirements beyond adenosine triphosphate production [33].

Glycine is synthesized through several pathways: (i) from glycolysis, mainly [34]; and (ii) from choline by the oxidation of choline to betaine, which is further demethylated to Gly. The major pathway for the intracellular metabolism of choline in breast cancer cells is by Gly production along with phosphatidylcholine synthesis [35,36]. Preclinical studies using animal models of basal-like and luminal-like breast cancer subtypes [37] showed increased Gly in the basal-like model relative to the luminal-like model [36,37]. It is known that the basal-like subtype of breast cancer has a poor prognosis, and hence increased Gly in breast cancer patients with a poor prognosis may be the result of altered glycolysis and/or metabolism of choline. These findings imply that the analysis of breast cancer tissue by MRS provides the metabolic state of a tumor, which provides additional information concerning the prognosis of the breast cancer.

### 2.3. In vivo MR Spectroscopy

As discussed earlier, the two most commonly utilized nuclei for investigating human tissues in vivo are ^1^H and ^31^P. In vivo MRS allows the detection of small molecules or metabolites. Generally, an in vivo MR spectrum is acquired from a localized ROI from a tumor so that contamination from external normal-appearing tissue is avoided. Localization of the ROI can be achieved by two methods. First is the single-voxel (SV) MR spectroscopy method which records the spectra from one ROI of the organ under investigation at a time. Second is the multi-voxel MR spectroscopy method (called MR spectroscopic imaging (MRSI), or chemical shift imaging (CSI)). In this method, MR spectra are recorded simultaneously from multiple voxels of the tumor and thereby map out the spatial distribution of the metabolites within the tissue.

In vivo ^1^H MRS shows the highest sensitivity and is the method of choice in most studies on breast cancer. It can detect metabolites that include resonances from lipids and choline-containing compounds, denoted as total choline (tCho). Generally, in vivo ^1^H MRS includes a prior MR imaging of the breast to localize the ROI of the tumor from which the MRS data is to be acquired. Normally the MR spectrum is acquired without and with water and fat suppression. Spectra obtained without any suppression provides information on the water and fat content of the breast tissues, while the suppressed spectrum gives the signal from tCho. The parameters derived from the ^1^H MR spectrum for characterizing the breast cancer malignancy are the water-to-fat (W/F) ratio and tCho peak.

**Figure 2:**
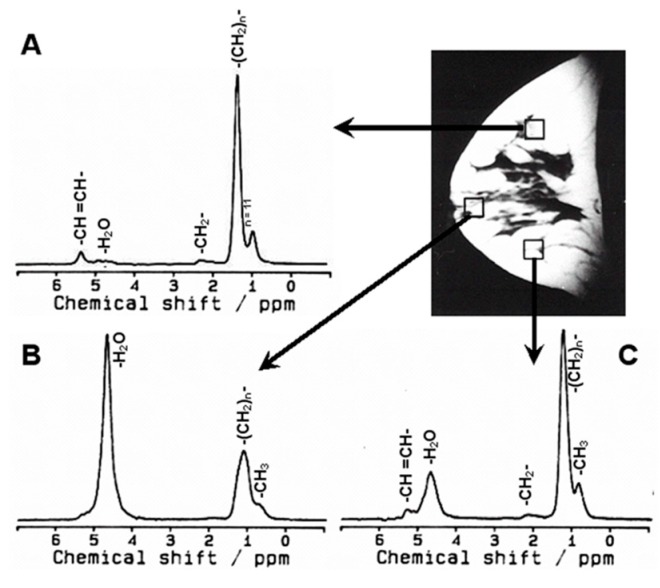
In vivo ^1^H MR spectra acquired at an echo time (TE) = 135 ms from three different voxel (8 mL) locations within the normal breast of a 31-year-old normal female volunteer: (**A**) upper quadrant; (**B**) para-areolar region; (**C**) lower quadrant (Reproduced with permission from Elsevier from Reference [39]).

However, it is important to understand the changes in biochemistry and physiology of the metabolism of normal breast tissue. Alterations in lipid composition and lipase activity accompany breast disease. MRS, especially ^1^H MRS, has been used to study lipid metabolism as it can detect changes in lipid profiles in many diseases. However, before the method is used for the study of changes during malignant transformation, it is necessary to understand the lipid profile of normal breast tissue. Breast tissue, being heterogeneous in nature, is also influenced by hormonal variation during the various phases of the menstrual cycle. Proliferative activity occurs naturally in normal breast tissue throughout the menstrual cycle. Changes in the lipid composition of normal breast parenchyma throughout the menstrual cycle have been documented by Dzendrowskyj et al. [38]. Sharma et al. [39] carried out in vivo ^1^H MRS from various regions of the normal breast tissue of volunteers, namely the para-areolar region and the upper and lower quadrants during five histological phases of the menstrual cycle (see Figure 2). The W/F ratio was calculated and no significant difference was observed in the values for the upper and the lower quadrants of the breast during various phases of the menstrual cycle. In the para-areolar region, the W/F ratio was significantly higher compared to the upper and the lower quadrants during all phases. This reflects the dependence of the W/F value on the amount of glandular and adipose tissue and the heterogeneous nature of the breast. These results indicate that any assessment of breast pathology using W/F values should be carried out carefully and taking into consideration the location of the tumor within the breast, the time of menstruation and normal breast metabolism [39]. A few studies have also reported the measurement of fatty acid composition of breast adipose tissue by ^1^H MRS at 3 T [40] and at 7 T [41,42].

**Figure 3:**
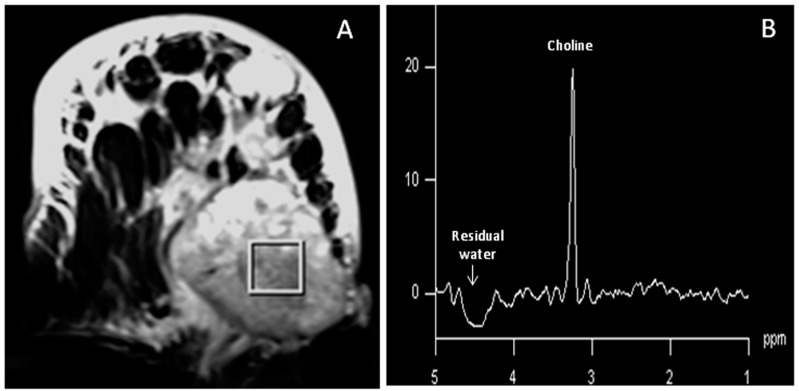
(**A**) T2-weighted, fat-suppressed axial image of a locally advanced breast cancer patient showing the location of a voxel of size 20 × 20 × 20 mm^3^ from which the corresponding ^1^H MR spectrum; (**B**) was obtained with water and lipid suppression (Reproduced with permission from John Wiley and Sons from Reference [55]).

In vivo ^1^H MR studies obtained with water and fat suppression (see Figure 3) have documented high levels of total choline-containing compounds (tCho) at 3.2 ppm (arising due to trimethylammonium head groups of total pool of water soluble choline-containing compounds) in malignant breast tissue compared to benign and normal breast tissues [42,43,44,45,46,47,48]. Further, it has been reported that early breast cancer patients showed a higher tCho concentration compared to locally advanced breast cancer patients [49]. tCho is a composite peak that contains resonance from several choline containing compounds like PCho, free Cho, and GPC and its increase during malignancy indicates increased synthesis of cellular membranes [50]. Higher levels of tCho is associated with the increased membrane synthesis required for the proliferation of the tumor. Choline kinase, which is involved in membrane biosynthesis and specifically the phospholipases that catalyze the degradation of phosphatidylcholine (PtdCho), is enhanced in tumor cells [8,9,51].

Glunde et al. have reported that the major component contributing to the tCho elevation in malignant cells is PCho [51]. The major phospholipid component of cell membranes is PtdCho, of which PCho is the breakdown product and a precursor. PCho can be produced by the phosphorylation of choline through ChoK, from hydrolysis of PtdCho, or indirectly through Phospholipase D [52]. Thus, the higher level of tCho detected by in vivo MRS may be indicative of membrane turnover [52], increased malignant potential [53], or activation of oncogenic signaling [54]. Aboagye et al. showed that malignant transformation was the cause of abnormal choline metabolism rather than cell proliferation [53].

**Figure 4:**
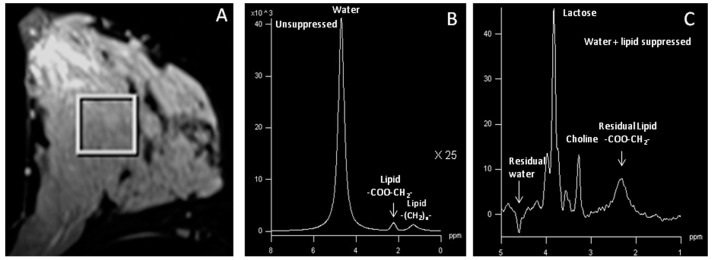
(**A**) T2-weighted, fat-suppressed axial image from the normal breast tissue of a lactating woman volunteer showing a voxel location of size 20 × 20 × 20 mm^3^; (**B**) corresponding ^1^H MR spectrum obtained without water and lipid suppression showing the water and lipid peaks; (**C**) ^1^H MR spectrum obtained with water and lipid suppression showing the residual water and lipid along with the tCho and the lactose peaks (Reproduced with permission from John Wiley and Sons from Reference [55]).

tCho has also been detected in the breast tissue of healthy volunteers, lactating women (see Figure 4), and benign lesions [43,49,55,56]. The concentration is low in normal breast tissue with values in the range of 0.1 to 1 mmol/kg [55,56,57]. In benign cases, the range of tCho concentration was between 0.04 to 2.70 mmol/kg, while in malignant breast tissue the value showed a wide range from 0.76 to 21.2 mmol/Kg [55,58,59,60,61]. Such a behavior may be attributed to the molecular variability and the spatially heterogeneous nature of breast cancer. In diffuse enhancement cases, tCho detection may be difficult because of intermingling of tumor cells with adipose tissue [62]. Infiltrating ductal carcinoma is the most commonly studied breast malignancy using ^1^H MRS. Data taken from other histological subtypes of breast cancer studied by MRS are very few, and an 82% sensitivity of detecting tCho has been reported in these cases (infiltrating lobular carcinoma [44,45], medullary carcinomas [43,57], mucinous carcinomas, [43,44], and adenoid cystic carcinoma [43]). These observations indicate that the metabolism is different in different breast tissues and may relate to tCho being associated with the aggressiveness of malignant tumors.

Few studies have evaluated the diagnostic accuracy of breast ^1^H MRS. Baltzer and Dietzel [63] analyzed 1198 lesions from 1183 patients and showed a high specificity of 88% but with a low sensitivity of 73%. Further, their meta-analysis revealed that the sensitivity showed a wide range from 42% to 100%. They also documented that there is no advantage of performing the MRS either at 3 T or at 1.5 T, nor whether it was performed prior to or after administration of the contrast agent. Cen and Xu have carried out a meta-analysis of the discrimination between malignant and benign breast lesions using single-voxel ^1^H MRS data and reported a pooled sensitivity of 71% with 85% specificity [64]. A meta-analysis of in vivo post-contrast ^1^H MRS for the differentiation of malignant and benign breast lesions has also been carried out [65] and the study showed a sensitivity of 74% and a specificity of 78%. In 2015, another meta-analysis evaluated the diagnostic performance of the ^1^H MRS using the signal to-noise ratio (SNR) of tCho resonance for the differentiation of benign versus malignant breast lesions [66]. Using a tCho SNR ≥ 2 as the cut-off for malignancy, the analysis showed a higher diagnostic accuracy with 74% sensitivity (range 69–77%) and 76% specificity (range 71–81%). Very recently, Sardanelli et al. [67] reviewed ^1^H MRS studies reported from 2010 to 2015 and carried out a meta-analysis. They showed that the pooled sensitivity of ^1^H MRS ranged from 71% to 74% with the specificity in the range of 75% to 84%, which is similar to the earlier study of Baltzer and Dietzel [63]. These results imply that the overall diagnostic accuracy of ^1^H MRS is low. The potential for using the tCho measurement for the assessment of tumor response in neoadjuvant chemotherapy has also been reported. No tCho or significantly reduced tCho in those patients who respond to treatment has been documented [68]. Results of an American College of Radiology Imaging Network (ACRIN) trial of the utility of ^1^H MRS for early assessment of breast cancer has also been reported recently [69].

**Figure 5:**
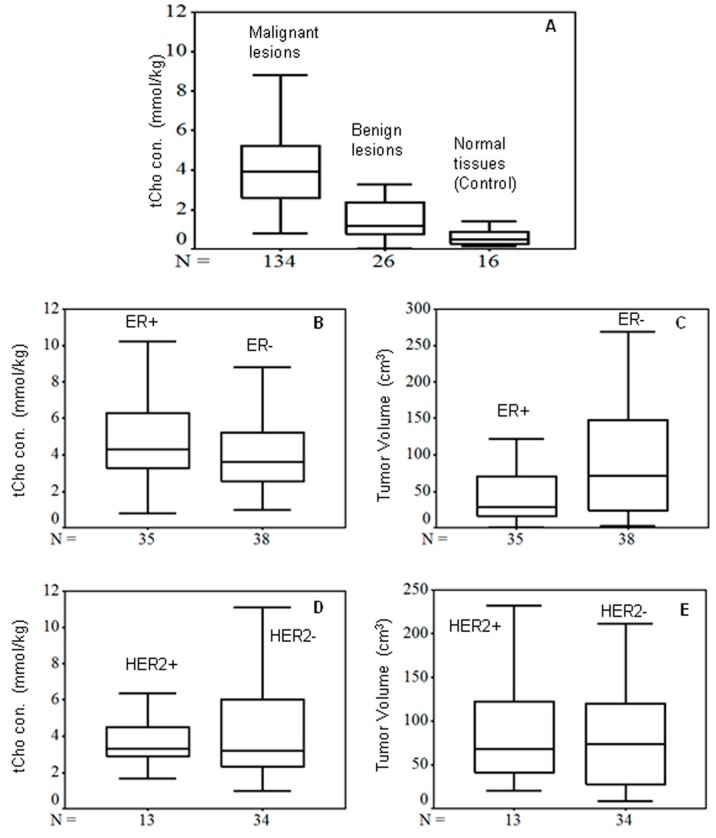
Box plot showing the comparison of (**A**) mean tCho concentration (mmol/kg) among malignant, benign and normal breast tissues (*p* < 0.01 among 3 groups); (**B**) variation of tCho concentration (mmol/kg) in estrogen receptor (ER) positive and negative breast cancer patients (*p* = 0.27); (**C**) tumor volume (cm^3^) in ER positive and negative breast cancer patients (*p* = 0.38); (**D**) tCho concentration (mmol/kg) in human epidermal growth factor receptor 2 (HER2) positive and negative patients (*p* = 0.16); (**E**) tumor volume with HER2 positive and negative breast cancer patients (*p* = 0.32). The midline across the boxes in the box plots represents the median value (Reproduced with permission from John Wiley and Sons from Reference [49]).

In a study from our laboratory, in vivo ^1^H MRS was carried out at 1.5 T on 128 locally advanced breast cancer patients (stage IIB, IIIA, IIIB and IIIC), 31 with early breast cancer (stage IIA), 38 with benign lesions, and 37 healthy female volunteers [49]. Our data showed that the tCho concentrations were significantly higher in early breast cancer compared to locally advanced breast cancer patients. Further, a statistically significant difference in tCho concentration was seen between patients at tumor stage IIA and those at stages IIIA and III (B&C). There are not many studies that have evaluated the accuracy of ^1^H MRS in tumor recurrence or re-staging. A tCho cut-off value of 2.54 mmol/kg was obtained for the differentiation between malignant and benign breast tissues while a cut-off of 1.45 mmol/kg was obtained for differentiating malignant from normal breast tissues [49]. tCho concentration showed no association with the ER, PR, or HER 2/neu status of patients when considered separately (see Figure 5). However, the tCho was lower in the triple negative in comparison to non-triple negative and triple positive breast cancer patients. These results indicate the complex molecular mechanisms of cell proliferation and the molecular heterogeneity of breast lesions as well as the differences in metabolism between various sub-types of breast tumors [49]. Recently, altered PtdCho metabolism in triple negative breast cancer progression has been documented in both human and experimental models [70].

The breast tissue architecture is maintained by steroid hormone and growth factor regulated pathways and disruption in any of these would lead to changes in the cell–cell interactions and the extracellular matrix adhesion leading to uncontrolled cell proliferation in breast cancer [71]. The Wnt/β-catenin pathway, among many molecular pathways, is characterized in breast cancer development [72]. Also, cyclin D1 is implicated in various malignant transformations and cancer progressions, including breast cancer [73,74]. In this direction, to understand the molecular mechanisms behind the elevated tCho level in breast cancer and its association with β-catenin and cyclin D, a study using in vivo MRS and enzyme-linked immunosorbent assay (ELISA) techniques was carried out in our laboratory [75]. Cytosolic and nuclear expressions of β-catenin and cyclin D1 were estimated using ELISA from 100 fractions isolated from malignant (*n* = 20), benign (*n* = 10) and non-involved breast tissues (*n* = 10) obtained after surgery. tCho levels are higher in malignant compared to benign tissues. Further, higher cytosolic and nuclear β-catenin expressions are observed in malignant tissues than in benign and non-involved breast tissues.

Both β-catenin and cyclin D1 expression was higher in the nucleus than in the cytosol within the malignant tissues. In the cytosol, cyclin D1 expression was higher in benign and non-involved tissue compared to malignant tissue. Our data showed a positive correlation of tCho with the cytosolic and nuclear expression of β-catenin and cyclin D1 in malignant tissues and a correlation between the nuclear expressions of both these proteins. Further, PR − patients showed higher cytosolic β-catenin expression than PR+ patients. Previous studies have shown a major role for activated choline phospholipid metabolism [72] and Wnt-mediated β-catenin signaling [76] pathways in cancer progression. However, for the first time, our study demonstrated a connection between these two pathways in breast cancer and a correlation between non-invasive biomarker, tCho and the Wnt/β-catenin pathway. This finding may have important implications in devising new diagnostic strategies for breast cancer patients. Furthermore, significant differences in choline and lipid metabolism and protein expression patterns have been documented between human breast cancer cells in cultures and tumors derived from these cultures [77], which highlights the influence of the tumor microenvironment on Cho and lipid metabolism.

### 2.4. Hyper-Polarized NMR

The examples that were presented in earlier sections provide evidence that ^1^H MRS measurements can be used to investigate tumor metabolism for diagnostic purposes, even though the clinical applications of MRS have been hampered by low sensitivity and consequently low spatial and temporal resolution, especially with the use of low-field MRI scanners (1.5 T or 3 T). In this direction, the nuclear spin hyperpolarization of ^13^C-labeled substrates using dynamic nuclear polarization (DNP) can increase the sensitivity of these substrates for detection by ^13^C MRS [78]. The use of DNP increases the SNR in the solution-state ^13^C MR experiments by 10^4^ to 10^5^-fold. However, the limitation is the short half-life of the polarization because of the short T1. For example, the half-life is only between 20s and 60s for [1-^13^C] pyruvate in vivo, which means that the hyperpolarized signal will last only for 2 min to 3 min. These timeframes mean that the substrate whose metabolism is to be imaged must be transferred from the polarizer and injected intravenously immediately. This means one must complete the experiment in 2 min to 3 min after administration of the hyperpolarized substance. In addition to ^13^C-labeled pyruvate, other molecules have been successfully hyperpolarized and their metabolic images have been obtained [79].

While the metabolite profiles obtained using ^1^H MRS and MRSI provide a static picture of tumor metabolism, imaging with hyperpolarized ^13^C-labeled substrates provides dynamic metabolic flux information. In fact, metabolic flux studies use isotope tracers like ^13^C, ^15^N, and ^2^H to track flow through metabolic pathways. The images are acquired at a relatively high spatial and temporal resolution and therefore provide an improved assessment of tumor behavior. Also, there is an increasing interest to study the intermediary metabolism of tumors which provides the energy required to sustain cancer cells. This energy is needed to maintain cation pumps and for cellular motility during metastasis. During cellular replication, tumor metabolism provides key metabolic precursors for DNA, protein, and lipid synthesis. Tracing the tumor intermediary metabolic pathways provides important information that is complementary to genomics for cancer diagnosis as well as prediction and early detection of a therapeutic response.

In vivo imaging of metabolites and their enzymatic conversion into other species, and the metabolic fluxes in central metabolic pathways, like glycolysis and the tricarboxylic acid cycle, has been reported [79]. Butt et al. have reported a correlation of tumor growth and the apparent pyruvate–lactate rate constant with disease progression with and without tamoxifen treatment in a mouse mammary tumor virus (MMTV)-PymT mouse model of breast cancer [80]. Also, Shestov et al. reported the validation of the the metabolic network model for analysis of flux through key pathways of tumor intermediary metabolism including glycolysis, the TCA cycle, and fatty acid biosynthesis and oxidation by using ^13^C isotopically labeled substrate MRS and LC-MS [81]. These were validated in melanoma and lymphoma cell models and show promise for studying tumor intermediary metabolism in other cancers like breast cancer, possibly in combination with the FDG-PET and hyperpolarized ^13^C MRS methods. Recently, an overview of the potential clinical role for metabolic imaging with hyperpolarized [1-^13^C] pyruvate has been reviewed [79].

### 2.5. ^31^P MR Spectroscopy

Using ^1^H MRS it is possible to detect the total level of choline containing compounds, but it is not possible to distinguish between the various phospholipid compounds like PCho, GPC, etc. In this direction, ^31^P MRS would be useful and can be carried out both in vivo and on breast cancer tissues obtained from surgery by in vitro NMR. Like ^1^H MRS, this technique also can be used to obtain tissue biochemistry by gathering information on the energy status of the tissue through the observation of various phosphate metabolites, intracellular pH, and the free cellular magnesium concentration. The energy metabolites that are detected using ^31^P MRS include nucleotide triphosphates (NTPs), phosphocreatine (PCr), and inorganic phosphate (Pi). In addition, membrane phospholipid resonances, like PME and PDE, are also observed. PME includes resonances from PCho and phosphoethanolamine, while PDE constitutes GPC and glycerophosphoethanolamine (GPE). These phosphate metabolites are involved in malignant transformation, tumor biology, and cell destruction through apoptotic mechanisms [82,83]. ^31^P MRS studies have shown increased levels of PME and PDE resonance that are metabolic characteristics of breast cancer. ^31^P MR has been shown to depend on the hormonal status of breast cancer patients [84]. However, it is still not suitable for the differentiation of malignant breast tumors from benign breast tumors [85]. The influence of the menstrual cycle on phosphate metabolites has also been reported in 7 female volunteers with regular menstrual cycles using 3D ^31^P MRSI at four time points during the menstrual cycle at 7 T. The study revealed that the phospholipids in the glandular breast tissue are not significantly affected by the menstrual cycle [86].

In recent years many improvements in the methodology of acquiring ^31^P MRS has been initiated, including carrying out investigations at high magnetic fields of 7 T. Early in vivo ^31^P MRS of human cancers reported a combined peak for PME and PDE resonances and the ratio of these peaks was used for assessing the therapeutic response of the tumor. However, with technological developments in radiofrequency (RF) coils, the use of high-field MR scanners etc., it is possible to resolve individual phosphate metabolites [87]. Klomp et al. demonstrated the ability to detect in breast cancer patients the various phospholipid metabolites like PCho, GPC, phosphoethanolamine, GPE and NTPs using in vivo localized ^31^P MRSI at 7 T [88]. The use of polarization transfer MRS for the improved detection of individual phosphate metabolites like PCho, phosphoethanolamine, GPC and GPE inorthotopic Michigan Cancer Foundation-7 (MCF-7), and MD Anderson – metastatic breast (MDA-MB-231) breast tumor xenografts at 9.4 T has also been reported [89].

Wijnen et al. have developed a method to improve the sensitivity for phosphate metabolites in vivo at 7 T with the use of proton observed phosphorus editing (POPE) [90]. Recently, the determination of the absolute concentrations of PCho, GPC and phosphoethanolamine using ^31^P MRSI has been reported [91]. The study showed that the reproducibility of the PME resonance using ^31^P MRSI was similar to that of tCho detection using ^1^H MRS at 7 T. Furthermore, the results indicated that the reproducibility of Pi, the sum of PMEs, and the sum of PDEs with ^31^P MRS was better than the detection of tCho using ^1^H MRS. An inverse correlation between the apparent diffusion coefficient and tumor grade, and between ^31^P MRS and mitotic count was reported using multiparametric MR (diffusion MRI and ^31^P MRS) data obtained at 7 T by Schmitz et al. [92].

The changes in membrane metabolism in breast cancer patients undergoing neoadjuvant chemotherapy using ^31^P MRSI has been documented [83,93]. Merchant et al. [83] investigated the phospholipid content in 43 malignant breast tissues obtained from patients undergoing surgery. The MR metabolic profile was correlated with the histology, clinical features, and hormone receptor status. They identified 14 phosphate metabolites from the NMR spectrum obtained at a ^31^P NMR frequency of 202.4 MHz. Of these, the mean mole percentage of sphingomyelin, phosphatidylcholine, phosphatidylserine, phosphatidic acid, phosphatidylglycerol, and alkylacylphosphatidylcholine predicted cellular infiltration, the infiltration type, elastosis, lymphatic invasion, perineural invasion, necrosis, and the estrogen receptor positivity [83]. This data showed that ^31^P MRS can be used for distinguishing the various pathologic subsets of breast cancer and their roles in cellular communication, regulation, and processes unique to malignant tissues. The ^31^P MR spectra at 7 T of patients undergoing therapy were monitored [93]. The spectral profile seen prior to therapy was different from that of the spectra from glandular breast tissue of healthy volunteers. However, during therapy, the spectral profiles of patients responding to treatment were seen to resemble those obtained from healthy volunteers. tCho, PME and PDE have also been shown to reverse upon successful treatment, and have been demonstrated to have the potential in predicting the response of the tumor to chemotherapy treatment [11,43,47,68,94,95,96,97].

## 3. Advantages and Disadvantages of MRS in the Study of Tissue Metabolism

The advantages of MRS over other imaging methods in the study of tissue metabolism are numerous. Even though conventional X-ray mammography is the primary imaging modality for breast cancer screening and diagnosis, its sensitivity for cancer detection is inconsistent with sensitivity between 69% and 90% and with variable specificity. The drawbacks of MRS include the radiation exposure and the fact that it is less sensitive and less specific in younger women with denser breast tissue. Ultrasonography, on the other hand, is a useful modality in the evaluation of dense breast tissue and in the differentiation of cysts from solid masses, abscesses, etc. However, it cannot be used as a screening method due to the difficulty of detecting micro-calcification in ductal carcinoma in situ, which has a variable false-negative rate. To date, computed tomography is not used for breast screening or diagnosis. It can be useful for contrast-enhanced lesions and lesions close to the chest wall. Further, computed tomography is generally advised for use in examining other parts of the body where the breast cancer has spread, such as the lymph nodes, lungs, liver, brain, or spine. Thus, all these are mainly diagnostic methods of cancer and none provide metabolic information at the molecular level for the investigation of tissue metabolism.

In view of the limitations, non-invasive methods such as MR imaging (MRI) and MR spectroscopy (MRS) have found greater application in the study of tissue metabolism. MRI is used for cancer detection and has a multiplanar imaging capability with a high-contrast resolution and uses no ionizing radiation. In vivo MRS allows noninvasive detection of the molecular composition of tissues and is a promising technique that provides biochemical information that is clinically valuable in the management of patients with breast disease. Further, it provides information on the physiological processes of malignant transformation and helps in understanding tumor metabolism through the measurement of endogenous metabolites. Moreover, addition of MRS to the routine breast MRI procedure improves specificity, which has been reported to be around 88% [68].

Even though MRS has great promise, there are few limitations. Generally, resonance from macromolecules like proteins and nucleic acids are not detected. Another issue is the difficulty of obtaining sufficiently well-resolved spectra because of the low field that is employed in most MR imaging (1.5 T or 3 T). Further, the detection of resonance from the potentially detectable metabolites is limited by their concentrations in the tissue. Thus, most in vivo MRS investigations have had long acquisition times (adds roughly another extra 10 min to 15 min to the routine MRI) and hence an increase in the overall patient scanning time. Specifically, breast ^1^H MRS in a routine clinical scenario is behind brain and prostate MRS. In breast MRS, typically a single peak at 3.22 ppm due to choline-containing compounds is detected, unlike brain or prostate MRS which are rich with metabolites. A low-quality MR spectrum is often seen, especially with breast ^1^H MRS, due to chest wall motion, improper shimming etc. Also, in most circumstances the need to use a slightly larger lesion size is required to detect a tCho resonance. Hence, the future investigations should focus on the development of optimized sequences capable of providing high SNR spectra, especially from small lesions and with short acquisition times. Standardization and quantification of metabolites in vivo is still not a routine procedure, which is yet another drawback. Thus, it is necessary to develop robust quantitative methods, to improve the design of coils with high sensitivity, and to evaluate the use of parallel imaging, better magnetic field shimming procedures, and standardization of post-processing spectral algorithms. Even though such limitations exist, in vivo MRS has been shown to be very useful to study of many human cancers [98,99].

## 4. Conclusions

In this review, several examples of the application of MRS in the study of breast cancer tissue metabolism have been presented. The literature consists of vast amount of excellent work on the study of metabolism in breast cancer, ranging from cells to live human tissues using NMR/MRS. It was not possible for us to review all of them, but the examples that are presented here show promise that NMR/MRS can be used as a noninvasive imaging tool for detecting metabolic biomarkers that can be used for diagnosing patients and monitoring their therapy.

The in vivo ^1^H MRS studies showed that the tCho concentration was higher in early breast cancer compared to locally advanced breast cancer patients. No association between the ER, PR and HER2 status of patients and tCho measurements was seen when considered separately. When all three molecular markers were taken into account in triple negative, non-triple negative, and triple positive groups, significant differences in the tCho concentration and age were observed. Recently, a correlation between tCho and the Wnt/β-catenin pathway was shown in breast cancer using in vivo MRS and ELISA. These observations indicate the complex molecular mechanisms of cell proliferation and the molecular heterogeneity of the breast lesions. Further, the changes seen in the lipid profile of breast tissue using ^1^H MRS showed that this is important in defining the stage of breast cancer, and gave an understanding of the cellular biochemistry and lipid metabolism associated with tumor development and progression. Further, this review also presented the application of in vitro HRMAS and hyper-polarized NMR in the study of breast tissue metabolism. In fact, in vivo MRS data are generally compared with both the ex vivo and in vitro NMR profiles of tissues to validate the clinical MR observations and, in fact, the techniques are complimentary to each other to obtain information regarding tissue metabolism.

The data from ^31^P MRS provides information on tissue bioenergetics through the observation of various resonances due to NTPs, Pi, etc. The results indicate that the increased PME level in breast cancers may be related to enhanced cell membrane synthesis, cellular growth, or nutrient availability and signaling by lipid hydrolysis. The NMR studies of tumor extracts, animal models, cell models, etc. show that some of the metabolites detected have the potential to serve as biomarkers and as indicators of therapy response.
